# Multiparametric Ultrasound (mpUS) of a Rare Testicular Capillary Hemangioma

**DOI:** 10.1155/2019/7568098

**Published:** 2019-12-28

**Authors:** Paul Spiesecke, Thomas Fischer, Carsten Stephan, Andreas Maxeiner, Bernd Hamm, Markus Lerchbaumer

**Affiliations:** ^1^Department of Radiology, Charité-Universitätsmedizin Berlin, Corporate Member of Freie Universität Berlin, Humboldt-Universität zu Berlin, and Berlin Institute of Health, Berlin, Germany; ^2^Department of Urology and Berlin Institute for Urologic Research, Charité-Universitätsmedizin Berlin, Corporate Member of Freie Universität Berlin, Humboldt-Universität zu Berlin, and Berlin Institute of Health, Berlin, Germany

## Abstract

Capillary hemangioma is a rare entity among testicular tumors. We demonstrate the case of an 18-year-old patient with palpatoric and sonographic conspicuous left testicle and negative serum tumor markers (*α*-fetoprotein, *β*-human chorionic gonadotropin, and lactate dehydrogenase). Ultrasound (US) imaging represented an isoechogenic lesion with high vascularization in both power Doppler and microflow imaging with central feeding artery. Both strain elastography and shear wave elastography demonstrated a stiff lesion compared to surrounding testicular tissue. While contrast-enhanced ultrasound (CEUS) clearly depicted high vascular load, time intensity curve (TIC) analysis was able to show shorter median transit time, higher peak enhancement, and higher wash-in area under the curve compared to regular testicular tissue. Histopathological examination revealed a lobular constructed and rich vascularized proliferation without cellular atypia and feeder vessels with positive reaction to CD34, CD31, CD99, and Vimentin. Proliferative activity was quantified to 3–5% by Ki-67 index. Two days after surgery, the patient could leave the hospital in subjective wellbeing. While histology remains the gold standard to make a precise diagnosis of capillary hemangiomas due to small case numbers and variety of this benign tumor, the combination of multiparametric US and clinical information may be a promising future tool in preoperative assessment.

## 1. Introduction

Capillary hemangioma is a rare entity among testicular tumors. Nevertheless, this benign vascular tumor is an important differential diagnosis since the most common malignant neoplasm in young men is testicular carcinoma, with increasing incidence [1]. Testicular capillary hemangioma seems to appear especially in young men as well, whereby this assumption should be considered with caution because of the small number of reported cases [2]. In addition to capillary form of testicular hemangioma, there are cavernous and epithelioid variants described as well. Multiparametric ultrasound (mpUS) means the combination of the established methods B-mode US, color-coded duplex sonography (CCDS), contrast-enhanced US (CEUS), and elastography [3]. Elastography measures the stiffness of tissue and can be distinguished in strain (longitudinal pressure generated by the examiner's compression of tissue beneath the probe) and shear wave elastography (SWE; impulse of the probe generating a transversal wave which velocity relates to local stiffness). CEUS uses the dynamic real-time character to depict vascularization and perfusion of a certain tissue or lesion in comparison to the surrounding tissue by evaluating wash-in and wash-out of a contrast agent. Overall, a variety of different sonographic parameters (multiparametric) can give in synopsis the diagnosis and may decrease operator dependency by providing quantitative results [3].

## 2. Case Description

We demonstrate the case of an 18-year-old patient who was admitted to our interdisciplinary US centre by an external urologist with palpatoric and sonographic conspicuous left testicle. Clinical examination revealed a caudal induration of the left testicle, which was nevertheless indolent. Epididymis and contralateral testicular parenchyma were inconspicuous. Serum tumor markers were negative: *α*-fetoprotein (*α*-AFP, 1.1 ng/ml), *β*-human chorionic gonadotropin (*β*-HCG, <0.1 U/l), and lactate dehydrogenase (LDH, 172 U/l).

**Figure 1:**
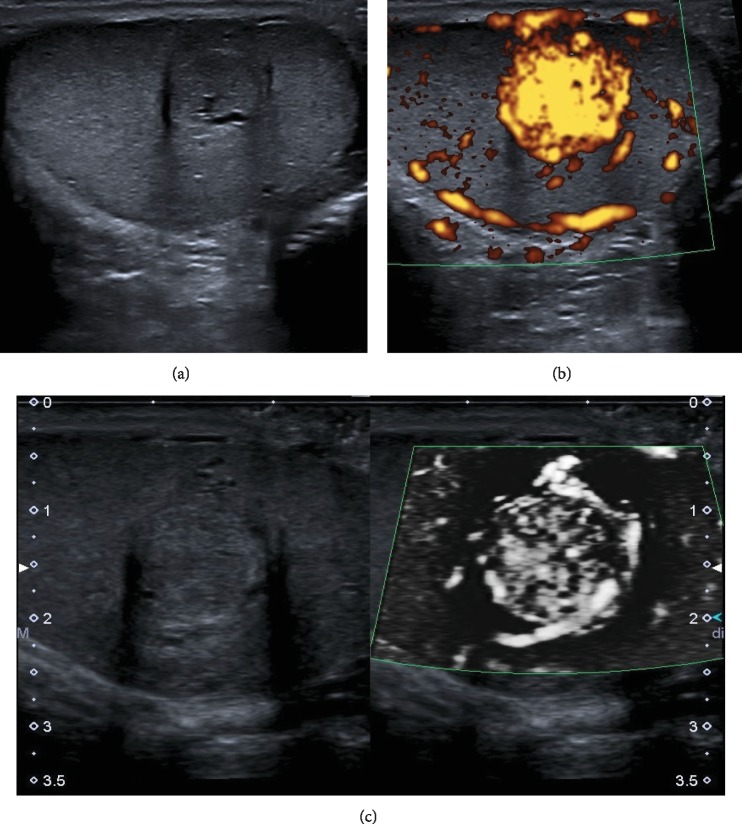
B-mode US, power Doppler imaging, and SMI of a capillary hemangioma. (a) Isoechogenic lesion in the central part of the left testicle with peripheral hypoechogenic rim (11 mm) and central feeder vessel. (b) Power Doppler imaging showed strong vascularization of the lesion compared to the surrounding tissue. (c) Monochromatic SMI in split screen mode determined stronger feeder vessels in the peripheral part of the lesion with high central vascular density. Abbreviations: US: ultrasound; SMI: superb microvascular imaging.

**Figure 2:**
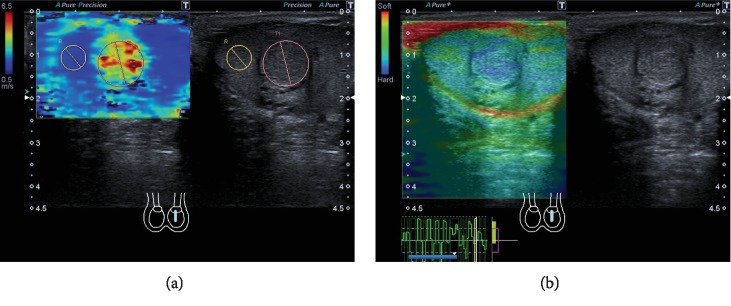
Elastographic assessment of the tumor lesion using 2D shear wave elastography (SWE) and strain elastography. (a) 2D shear wave elastography demonstrates higher and more inhomogenous stiffness of the tumor lesion (4.24 m/s) compared to the surrounding testicular tissue (1.49 m/s) with a SWE ratio of 2.85. (b) Tumor lesion represents blue (hard) in color-coded strain elastography compared to the surrounding testicular tissue (green-blue) presented by less compression capability indicating higher stiffness of the tumor. Abbreviations: SWE: shear wave elastography.

B-mode ultrasound of the left testicle showed an isoechogenic, round-shaped lesion in the central left testicle up to 11 mm diameter with a hypoechogenic peripheral rim (Figure 1(a)). Power Doppler imaging (PDI) and microflow imaging (e.g., SMI (superb microvascular imaging)) depicted hypervascularization that clarified the finding of a vascular tumor (Figures 1(b) and 1(c)). While PDI only demonstrated strong vascularization, SMI was able to show bigger feeder vessels in the central part of the lesion with high density of smaller central vessels around (Figure 1(c)). Strain elastography demonstrated a harder lesion compared to the surrounding testicular tissue, while the lesion had a higher stiffness (up to 6.5 m/s) in 2D SWE compared to regular testicular tissue (0.5 m/s) (Figure 2). Furthermore, stiffness of the lesion was less homogenous than the regular surrounding tissue.

CEUS examination was performed and interpreted by a single high-experienced radiologist with more than fifteen years' experience in CEUS (EFSUMB level 3) using a high-end ultrasound system (Aplio i500, Canon, Otawara, Japan) with a linear broadband transducer (i14L5; Canon, Otawara, Japan). B-mode US was optimized using spatial compounding, frequency-based compounding, ApliPure™ level 5, differential Tissue Harmonic Imaging (dTHI)©, and Precision Imaging© with level 4 Speckle Reduction (SR). CEUS was performed at 9 MHz, 10 fps configured with a very low MI (0.07) to avoid early microbubble destruction. A bolus of 2.4 ml of ultrasound contrast agent (SonoVue®, Bracco Imaging, Milan, Italy) was injected up to three times.

**Figure 3:**
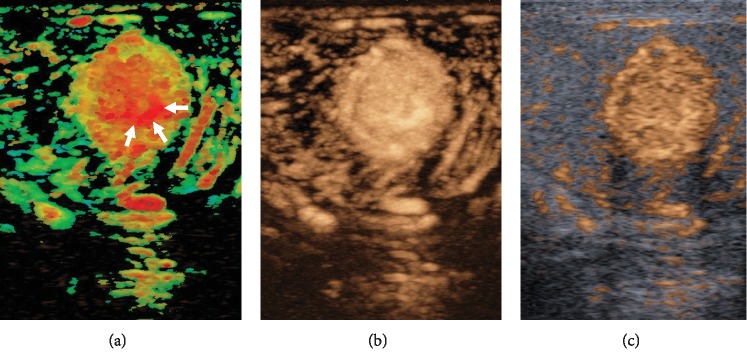
Arrival time imaging (ATI) and contrast-enhanced ultrasound (CEUS). (a) Parametric ATI depicted short arrival time in the central part of the tumor with feeder artery within 2 seconds (arrows; color-coded in red). (b, c) Accumulation mode and B-mode/CEUS image fusion demonstrates high peak intensity of the tumor compared to the surrounding testicular tissue. Abbreviations: ATI: arrival time imaging; CEUS: contrast-enhanced ultrasound.

**Figure 4:**
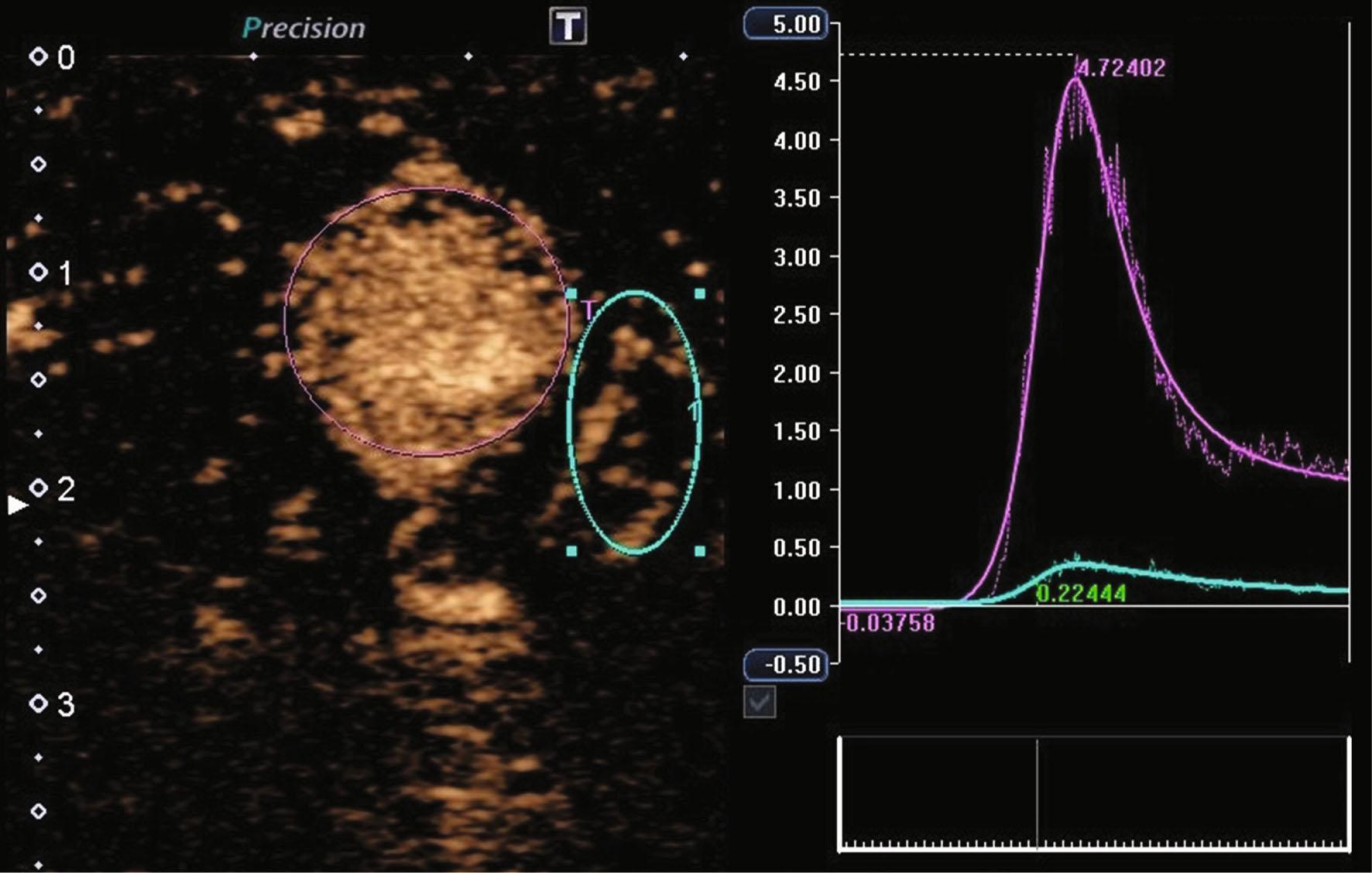
Time intensity curve (TIC) measurement using a ROI in tumor lesion and regular testicular tissue. TIC measurements demonstrate a higher peak intensity, shorter mean transit time, and higher wash-in AUC of the tumor lesion (purple ROI) compared to regular surrounding tissue (blue ROI). Abbreviations: TIC: time intensity curve; ROI: region of interest; AUC: area under the curve.

The hypervascularized lesion showed early and strong contrast enhancement compared to the surrounding tissue. Arrival time imaging depicted a shorter arrival time feeder vessel with short central filling of the lesion due to high vessel density (Figure 3). In comparison to the surrounding tissue, time intensity curve (TIC, Table 1) measurement showed a clearly higher peak intensity (45.6 vs. 3.4), shorter mean transit time (7.0 vs. 10.1 sec), and higher wash-in area (121.3 vs. 9.1), while time to peak showed no difference (4.4 vs. 4.2 sec) (Figure 4).

The patient received enucleation and intraoperative incision by highly experienced surgeons. Histopathological examination revealed a lobular constructed and rich vascularized proliferation without cellular atypia and feeder vessels. Immunohistology revealed positive reaction to CD34, CD31, CD99, and Vimentin. Moreover, there was a significant capillary growth form in the tumor's periphery between preexisting testicular tubules. The capillaries were continuously endowed with pericytes and showed partial expression of WT-1. Proliferative activity was quantified to 3–5% by Ki-67 index. Two days after surgery, the patient could leave the hospital in subjective wellbeing.

## 3. Discussion

The high vascularization in our case could have been shown congruous with PDI, SMI, and CEUS. Since hyperenhancement of a testicular lesion had a positive predictive value of 97.4% for neoplasia [4], the sonographic findings in our case indicated potential malignancy. Besides, the sonographic findings with central feeding vessels could allow a differentiation to benign Leydig cell tumor, which is suggested by a short filling time or by a circumferential vessel with a rapid centripetal filling [5]. The wash-in of the hemangioma was higher and more rapid than the surrounding tissue, as demonstrated by a shorter time to peak and higher peak enhancement and wash-in rate. The homogenous enhancement could be thought to be due to a high blood flow velocity and an increased microvessel density, while tumors like seminomas or burn-out tumors often show areas of less enhancement due to necrosis [6]. Furthermore, malignant lesions were more often described to be hypoechogenic compared to the surrounding tissue [7]. Contrary to our results, capillary hemangioma has also been demonstrated as a hypoechogenic lesion with low signal in CCDS [8].

In general, CEUS can be a very helpful method to investigate microvascularization of testicular lesions. CEUS may be valuable in the assessment of contrast enhancement in small intratesticular masses (<5 mm) where color-coded duplex sonography comes to its limits because small testicular tumors may appear avascular. Since it is possible to distinguish solid and cystic or burn-out tumors, previous studies found no key finding in enhancement pattern to differentiate between benign and malignant tumors especially in small lesion (<10 mm). Furthermore, clinical presentation may be similar between testicular hemangioma and malignant testicular lesions with slight testicular pressure leading to pain.

CEUS has advantages over contrast-enhanced magnetic resonance imaging (MRI) including unmatched temporal resolution due to continuous real-time imaging [9]. US remains the primary imaging method in unclear testicular pain or incidental finding of testicular lesion. Therefore, CEUS is a fast and easy tool to elucidate unclear B-mode findings. Moreover, it is a safe tool because CEUS examinations do not use radiation and the contrast agent applied has no renal, thyroid, or cardiac toxicity.

While orchiectomy is recommended as the method of choice, enucleation and intraoperative incision showed a benign stroma tumor in this case, so the rest of the organ could remain. Nevertheless, histology remains the gold standard to make a precise diagnosis of a testicular tumor. Due to small case numbers and variety of this benign tumor, the combination of contrast-enhanced ultrasound, elastography, and clinical information (tumor markers) may be a promising future tool for treatment planning to avoid unnecessary orchiectomy especially in young patients.

## Figures and Tables

**Table 1:** Time intensity curve analysis using a ROI within the hemangioma and surrounding testicular tissue.

TIC parameter	Hemangioma	Testicular tissue
PI (1.0*E*-5 AU)	45.6	3.4
TTP (s)	4.2	4.4
MTT (s)	7.0	10.1
Slope (1.0*E*-5 AU/s)	13.0	1.0
Area (1.0*E*-5 AU/s)	2007.9	130.0
AreaWI (1.0*E*-5 AU·s)	121.3	9.7
AreaWO (1.0*E*-5 AU·s)	1886.6	120.3

ROI within the capillary hemangioma demonstrated higher PI, shorter MTT, higher slope, and higher wash-in/wash-out rates compared to regular testicular tissue. Abbreviations: ROI: region of interest; PI: peak intensity; TTP: time to peak; MTT: mean transit time; AreaWI: area wash-in; AreaWO: area wash-out.
